# Synthesis and Anti-Intestinal Nematode Activity of Variously Substituted Benzonaphthyridine Derivatives

**DOI:** 10.3390/molecules16021593

**Published:** 2011-02-14

**Authors:** Li-Ping Duan, Ai-Dan Wen, Ning-Bo Wu, Yi Tao, Hao-Bing Zhang

**Affiliations:** Key Laboratory of Parasitology and Vector Biology at National Institute of Parasitic Diseases, Chinese Center for Disease Control and Prevention, WHO Collaborating Centre for Malaria, Schistosomiasis, and Filariasis, National Institute of Parasitic Diseases, Shanghai 200025, China

**Keywords:** benzonaphthyridine, C=N linkage, *Nippostrongylus brazilliensis*

## Abstract

A series of benzonaphthyridine derivatives bearing the C=N linkage moiety were designed and synthesized. The structures of all the newly synthesized compounds were identified by elemental analysis, ^1^H-NMR, ^13^C-NMR and MS. Their anti-intestinal nematode activities against *Nippostrongylus brazilliensis* were evaluated *in vivo* by an oral route in male rats. Among these compounds, at concentrations of 10 mg/kg of rat, the compound 7-chloro-2-methoxy-10-(4-(4′-(1*H*-indol-5′-yl)methylene)aminophenyl)-amino-benzo[b][1,5] naphthyridine (**4n**) produced the highest activity, with 80.2% deparasitization. These compounds may find usefulness in the discovery and development of new anti-intestinal drugs.

## 1. Introduction

Intestinal parasitic nematodes or roundworms infect over 1 billion people in tropical countries. According to a 2005 report by the World Health Organization (WHO), approximately 0.807–1.221 billion humans have *ascariasis,* 604–795 million have *trichuriasis,* and 576–740 million have hookworm infections worldwide [1]. Despite their harmfulness, few drugs for dealing with them exist [2,3,4]. Tribendimidine has good single-dose efficacy against some roundworm parasites [5], and in studies [6,7,8] tribendimidine showed high efficacy against *Nippostrongylus braziliensis*, *Necator americanus, Ancylostoma caninum,* and *Toxocara canis.* Furthermore, tribendimidine can continue to be effective in areas where albendazole has become less so due to resistance. This has been attributed to the presence of a toxophoric C=N linkage [5]. In our research group, we have been interested in studying the design, synthesis, and biological activity of compounds, especially heterocyclic compounds [9,10,11,12,13,14]. During the last 30 years a large number of derivatives belonging to the general benzonaphthyridine class have been prepared and evaluated extensively as antimalarial, antileishmanial and antitrypanosomal compounds [15,16,17,18,19]. This inspired us to assume that benzo[b][1,5]-naphthyridine derivatives incorporating a Schiff base might have some improved or different biological activities. In the present work, we describe the synthesis and results of some preliminary *in vivo* biological tests of 15 different novel benzonaphthyridine derivatives bearing a C=N linkage moiety.

## 2. Results and Discussion

### 2.1. Synthesis and Characterization of substituted benzo-naphthyridine derivatives (**4a-4o**)

**Scheme 1:**
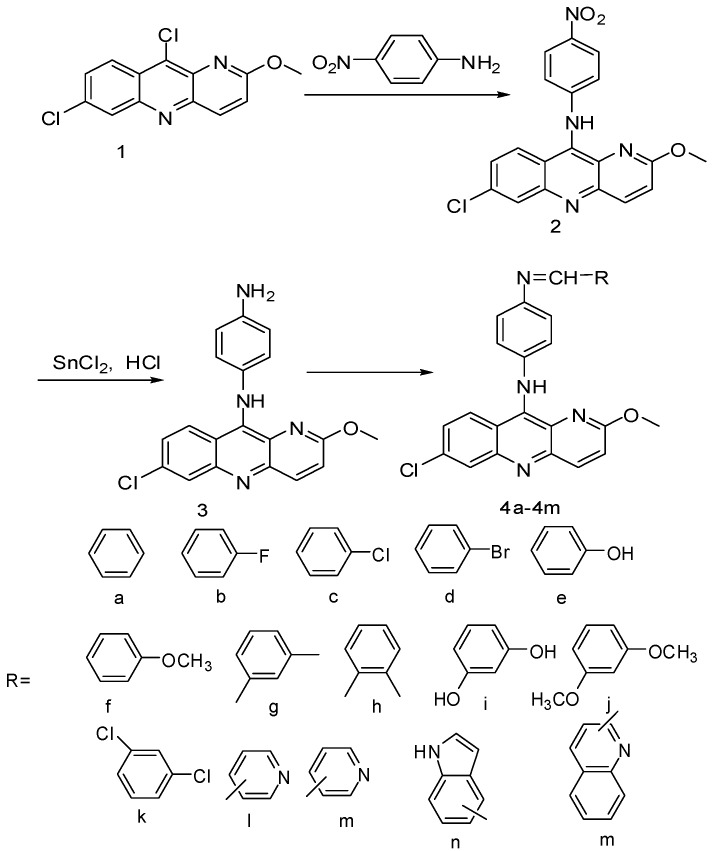
Synthesis of compounds **4a-4o**.

The synthetic route to the target compounds **4a-4o **is shown in Scheme 1. Benzo[b][1,5]-naphthyridine derivative **1** had already been described [20]. 

Direct nucleophilic substitution of chlorine at the 10 position of the benzo[b][1,5]-naphthyridine ring by *p*-nitroaniline afforded desired chloride **2** in good yield. The subsequent reduction of **2** using SnCl_2_ as reducing agent led to the key intermediate **3**. Then **3 **and different aromatic aldehydes were mixed in boiling ethanol affording the target compounds **4a–4o**, which were recrystallized twice from ethanol to give satisfactory yields of 35–50%, respectively. Compounds **4a–4o** were characterized by elemental analysis, ^1^H-NMR, ^13^C-NMR and MS. All results were in full agreement with the proposed structures. For example, the ^1^H-NMR spectrum of compound **4b **showed a singlet at 4.18 ppm (OCH_3_), singlet at 8.45 ppm (NH) and a singlet at around δ = 8.40 due to one proton of the Schiff base. This was also confirmed by ^13^C-NMR spectrum, which showed C=N signals at 160.71 ppm, moreover, the ^13^C-NMR spectrum showed δ 53.90(OCH_3_), 159.73, 154.89, 148.65, 148.02, 146.56, 143.71, 142.92, 142.50, 142.20, 140.49, 136.62, 135.11, 129.95, 129.09, 128.87, 127.76, 127.71, 126.60, 124.51, 122.58, 122.18, 119.42, 118.59，115.76 (Ar-C), all consistent with its proposed structure. The elemental analyses results were in good agreement with those calculated for the suggested formula. Further confirmation for the structure of compound **4b** was obtained by MS, which showed the expected [M+1]^+ ^ion at 457.27. Additionally, the melting points are sharp, suggesting the purity of these compounds.

### 2.2. Anti-intestinal nematode activity

From Table 1, we can see that some of compounds showed significant anti-intestinal nematode activity in a two-day *in vivo* test in rats. At concentrations of 10 mg/kg of rat, compound **4n **produced the highest activity against *Nippostrongylus brazilliensis*, with 80.3% deparasitization. 

**Table 1:** Effect of benzo[b][1,5] naphthyridine derivatives in treatment of rats infected with NbL3 for 11 days **(4a-4o)**.

Compound	Dose(mg/kg)	Number of rats	Worm recovery		
			Expelled	Total	Reduction (%)
**Albendazole**	10	5	245	248	98.8
**4a**	10	5	114	543	21.0
**4b**	10	5	75	297	25.2
**4c**	10	5	74	322	23.0
**4d**	10	5	48	237	20.2
**4e**	10	5	100	426	23.4
**4f**	10	5	75	269	27.9
**4g**	10	5	68	326	20.8
**4h**	10	5	69	287	24.0
**4i**	10	5	82	306	26.8
**4j**	10	5	89	370	24.0
**4k**	10	5	79	310	25.4
**4l**	10	5	95	189	50.2
**4m**	10	5	101	197	51.2
**4n**	10	5	215	268	80.2
**4o**	10	5	176	248	70.9

Expelled: number of expelled worms; Total: total worm number; Reduction: worm burden reduction.

To investigate the role of substituent effects on activity, a variety of substituents could be introduced on the phenyl ring. The F, OCH_3_, OH, Cl and Br-substituted derivatives all have almost the same activity as **4a**. Furthermore, disubstituted derivatives also appeared to have similar potency as the monsubstituted phenyl analogues. For instance, the replacement of the 4**′**-F in 4**′** by a hydroxyl group led to only slightly less potency, and compound **4f** inhibited *Nippostrongylus brazilliensis* with a value similar to that of the 2,4-disubstituted analogue **4j**. In contrast, derivatives **4g** and **4l **were slightly less active than the corresponding isomers **4h** and **4m**. On the other hand, replacement of the phenyl ring by a pyridyl ring, indole group, or quinoline group resulted in increased effects, suggesting a preference for hetero-aromatic rings in maximizing activity. This indicated that indole group was the most important group involved in this activity. The quinoline group also seems to influence this activity, while the pyridyl group appeared to give only a small contribution. Because compound **4n **displayed anti-intestinal nematode potency that was comparable to albendazole (10 mg/kg), further anti-intestinal nematode activity assayd were carried out for compound **4n**. It was found that at concentrations of 30 mg/kg of rat, **4n **produced the highest activity against *Nippostrongylus brazilliensis* with 98.8% effectiveness, which implies further possibilities for the development of new anti-intestinal drugs.

## 3. Experimental

### 3.1. Materials and reagents

All solvents and other reagents were of high purity (Aldrich and Sigma) and were used without further purification. Elemental analysis was performed on a PE-2400 Elemental Analyzer, the C, H and N analysis were repeated twice. ^1^H-NMR and ^13^C-NMR spectra were obtained in CDCl_3_ with TMS as internal standard on a Bruker AM-400 spectrometer. Chemical shifts were reported as ppm. Mass spectra were measured on a HP 1100 LC-MS spectrometer. Melting points were determined by an BÜCHI melting point B-540 apparatus and are uncorrected.

### 3.2. Synthesis of 7-chloro-2-methoxy-10-aminobenzo[b][1,5]naphthyridine (**2**)

A solution obtained dissolving equimolar amounts of the starting compound 7-chloro-2-methoxy-10-chloro-benzo[b][1,5]naphthyridine (**1**) and *p*-nitroaniline in absolute ethanol (150 mL) was refluxed for 2.5–3 h until the starting materials disappeared (TLC, ethyl acetate/*n*-hexane 1:4). On standing and cooling a red-orange precipitate formed which was filtered off and dried (70–80% yields). Purification was accomplished by repeated crystallization from ethanol. LC-MS: [M+1]^+ ^381.17.

### 3.3. Synthesis of 7-chloro-2-methoxy-10-(4-aminophenyl)-aminobenzo[b][1,5] naphthyridine(**3**)

Compound **2** (3.80 g, 0.01 mol) was added to a previously prepared solution of glacial acetic acid (40 mL) containing SnCl_2_ (15.3 g, 0.08 mmol) and aerated with hydrogen chloride. The reaction mixture was stirred for 1 h. The precipitated solid was collected by filtration and washed with water. Crystallization from acetic acid gave **3** as red–orange needles: 3.46 g (98.8%); LC-MS: [M+1]^+ ^351.29.

### 3.4. General procedure for the synthesis of Schiff bases **4a-4o**

To a mixture of **3 **(0.005 mol) and substituted aromatic aldehyde (0.005 mol) in absolute ethanol (40 mL) was added few drops of glacial acetic acid. Then the mixture was refluxed on water bath for 5–6 h. The excess of solvent was distilled off, poured onto ice cold water. The separated solid was filtered, washed and recrystallized from ethanol.

*7-Chloro-2-methoxy-10–(4-benzylideneaminophenyl)-aminobenzo[b][1,5]naphthyridine* (**4a**). Yield 35%, m.p. 180.2–181.3 °C. ^1^H-NMR δ: 8.41 (s, 1H, Ar-NH), 8.37(s, 1H, CH=N), 8.24–6.82 (m, 14H, Ar-H), 4.16 (s, 3H,OCH_3_); ^13^C-NMR δ: 160.20, 159.62, 154.70, 149.00, 148.12, 146.45, 143.71, 142.00, 140.50, 137.63, 131.65, 130.86, 130.78, 129.24, 124.78, 123.16, 123.10, 122.17, 122.16, 121.76, 119.28, 119.16, 115.78, 114.44, 113.15, 53.69; Anal. Calcd. for C26H19 Cl N4O (439.82): C 71.15, H 4.36, N 12.77; found C 71.16, H 4.38, N 12.77.

*7-Chloro-2-methoxy-10–(4-(4′-fluorobenzylidene)aminophenyl)-aminobenzo[b][1,5]naphthyridine* (**4b**)*.* Yield 40%, m.p. 191.6–191.8 °C. ^1^H-NMR δ: 8.45 (s, 1H, Ar-NH), 8.40 (s, 1H, CH=N), 8.29–7.02 (m, 13H, Ar-H), 4.18 (s, 3H,OCH_3_); ^13^C-NMR δ: 160.71, 159.73, 154.89, 148.65, 148.02, 146.56, 143.71, 142.92, 142.50, 142.20, 140.49, 136.62, 135.11, 129.95, 129.09, 128.87, 127.76, 127.71, 126.60, 124.51, 122.58, 122.18, 119.42, 118.59, 115.76, 53.90; Anal. Calcd. for C26H18 ClF N4O (457.27): C 68.39, H 4.00, N 12.07; found C 68.39, H 4.00, N 12.07.

*7-Chloro-2-methoxy-10–(4-(4′-chlorobenzylidene)aminophenyl)-aminobenzo[b][1,5]naphthyridine* (**4c**). Yield 42%, m.p. 193.4–194.1 °C. ^1^H-NMR δ: 8.45 (s, 1H, Ar-NH), 8.40 (s, 1H, CH=N), 8.25–6.98 (m, 13H, Ar-H), 4.16 (s, 3H,OCH_3_); ^13^C-NMR δ: 160.83, 159.80, 154.70, 149.79, 148.32, 144.40, 143.87, 142.21, 140.36, 138.06, 132.56, 131.45, 129.65, 126.09, 124.87, 123.76, 122.67, 122.48, 121.69, 119.20, 119.17, 117.09, 116.47, 115.20, 53.80 ; Anal. Calcd. for C26H18 Cl_2_ N4O (474.25): C 65.97, H 3.83, N 14.98; found C 65.99, H 3.83, N 14.98.

*7-Chloro-2-methoxy-10–(4-(4′-bromobenzylidene)aminophenyl)-aminobenzo[b][1,5]naphthyridine* (**4d**). Yield 37%, m.p. 188.6–191.0 °C. ^1^H-NMR δ: 8.47 (s, 1H, Ar-NH), 8.40(s, 1H, CH=N), 8.25–7.00 (m, 13H, Ar-H), 4.17 (s, 3H,OCH_3_); ^13^C-NMR δ: 160.83, 159.60, 149.71, 148.38, 144.84, 143.89, 142.41, 140.40, 138.96, 132.66, 131.95, 129.75, 126.49, 125.67, 124.86, 123.69, 123.68, 120.69, 119.29, 119.20, 118.89, 117.57, 116.27, 115.48, 53.90 ; Anal. Calcd. for C26H18 BrCl N4O (519.21): C 60.31, H 3.50, N 10.82; found C 60.31, H 3.51, N 10.85.

*7-Chloro-2-methoxy-10–(4-(4′-hydroxylbenzylidene)aminophenyl)-amino-benzo[b][1,5] naphthyridine* (**4e**). Yield 39%, m.p. 201.3–201.5 °C. ^1^H-NMR δ: 8.44 (s, 1H, Ar-NH), 8.20 (s, 1H, CH=N), 8.28–6.84 (m, 13H, Ar-H), 5.50 (s, 1H,OH), 4.18 (s, 3H,OCH_3_); ^13^C-NMR δ: 160.83, 160.21, 159.74, 149.68, 148.45, 144.91, 143.56, 142.35, 138.95, 132.45, 130.68, 129.87, 127.49, 125.86, 123.89, 123.70, 123.45, 120.69, 119.34, 119.08, 118.54, 116.60, 116.27, 115.50, 114.56, 59.62 ; Anal. Calcd. for C26H19ClN4O_2_ (455.28): C 64.43, H 4.05, N 11.58; found C 64.43, H 4.05, N 11.58.

*7-Chloro-2-methoxy-10–(4-(4′-methoxylbenzylidene)aminophenyl)-aminobenzo[b][1,5] naphthyridine* (**4f**). Yield 45%, m.p. 170.8–171.1 °C. ^1^H-NMR δ: 8.87 (s, 1H, Ar-NH), 8.40(s, 1H, CH=N), 8.20–6.68 (m, 13H, Ar-H), 4.18 (s, 3H,OCH_3_), 3.82 (s, 3H,OCH_3_); ^13^C-NMR δ: 160.73, 159.23, 149.91, 148.98, 145.82, 143.90, 142.53, 138.96, 132.70, 131.90, 129.86, 127.31, 126.77, 125.77, 123.54, 123.25, 121.99, 120.29, 119.78, 118.96, 116.78, 116.20, 106.48, 103.76, 54.60, 53.82; Anal. Calcd. for C27H21Cl N4O_2_ (419.18): C 69.15, H 4.51, N 11.95; found C 69.16, H 4.51, N 11.96. 

*7-Chloro-2-methoxy-10–(4-(2′,4′-dimethylbenzylidene)aminophenyl)aminobenzo[b][1,5] naphthyrid-ine* (**4g**). Yield 46%, m.p. 161.1–161.9 °C.^ 1^H-NMR δ: 8.80 (s, 1H, Ar-NH), 8.40 (s, 1H, CH=N), 8.39–6.78 (m, 12H, Ar-H), 4.18 (s, 3H,OCH_3_), 2.55(s, 3H, CH_3_), 2.35 (s, 3H, CH_3_); ^13^C-NMR δ: 160.61, 158.34, 149.11, 148.74, 143.12, 142.87, 141.49, 140.81, 140.48, 138.58, 135.18, 131.88, 131.48, 125.77, 128.86, 128.41, 127.91, 127.26, 126.75, 124.28, 122.79, 122.15, 119.37, 115.46, 53.79, 21.57,19.47; Anal. Calcd. for C28H23Cl N4O (467.29): C 69.10, H 5.34, N 11.49; found C 69.18, H 5.34, N 11.43.

*7-Chloro-2-methoxy-10–(4-(2′,3′-dimethylbenzylidene)aminophenyl)-aminobenzo[b][1,5] naphthyrid-ine* (**4h)**. Yield 48%, m.p. 125.1–125.7 °C. ^1^H-NMR δ: 8.45 (s, 1H, Ar-NH), 8.40(s, 1H, CH=N), 8.20–7.01 (m, 12H, Ar-H), 4.18 (s, 3H,OCH_3_), 2.34(s, 6H, CH_3_); ^13^C-NMR δ: 160.62, 158.34, 149.11, 148.74, 143.10, 142.80, 141.48, 140.80, 140.47, 138.56, 135.08, 131.78, 131.48, 125.77, 128.86, 128.41, 127.90, 127.26, 126.70, 124.28, 122.78, 122.05, 119.37, 115.46, 53.89, 21.47,19.37; Anal. Calcd. for C28H23Cl N4O (467.28): C 71.87, H 4.62, N 11.68; found C 71.87, H 4.62, N 11.68.

*7-Chloro-2-methoxy-10–(4-(2′,4′-dihydroxylbenzylidene)aminophenyl)-aminobenzo[b][1,5]naphthyr-idine* (**4i**). Yield 44%, m.p. 232.1–232.9 °C. ^1^H-NMR δ: 8.45 (s, 1H, Ar-NH), 8.40(s, 1H, CH=N), 8.20–7.01 (m, 12H, Ar-H), 4.18 (s, 3H,OCH_3_), 1.44(s, 2H, OH); ^13^C-NMR δ: 160.63, 158.38, 149.16, 148.77, 143.13, 142.80, 141.48, 140.83, 140.49, 138.58, 135.68, 131.80, 131.56, 125.79, 128.89, 128.43, 127.92, 127.36, 126.78, 124.59, 122.78, 122.16, 119.39, 115.48, 53.91; Anal. Calcd. for C26H19Cl N4O_3_ (471.27): C 66.07, H 3.76, N 11.50; found C 66.07, H 3.76, N 11.50.

*7-Chloro-2-methoxy-10–(4-(2′,4′-methoxylbenzylidene)aminophenyl)-aminobenzo[b][1,5] naphthyr-idine* **(4j)**. Yield 45%, m.p. 160.2–161.8 °C. ^1^H-NMR δ: 8.89 (s, 1H, Ar-NH), 8.40 (s, 1H, CH=N), 8.20–6.55 (m, 12H, Ar-H), 4.18 (s, 3H,OCH_3_), 3.83 (s, 3H,OCH_3_), 3.82 (s, 3H,OCH_3_); ^13^C-NMR δ: 160.73, 159.23, 149.91, 148.98, 145.82, 143.90, 142.53, 138.96, 132.70, 131.90, 129.86, 127.31, 126.77, 125.77, 123.54, 123.25, 121.99, 120.29, 119.78, 118.96, 116.78, 116.20, 115.48, 114.76, 54.60, 53.82, 53.80; Anal. Calcd. for C28H23Cl N4O_3_ (499.29): C 66.21, H 5.19, N 10.52; found C 66.21, H 5.19, N 10.52. 

*7-Chloro-2-methoxy-10–(4-(2′,4′-dichlorobenzylidene)aminophenyl)-aminobenzo[b][1,5] naphthyr-idine* (**4k**). Yield 39%, m.p. 220.1–221.2 °C. ^1^H-NMR δ: 8.59 (s, 1H, Ar-NH), 8.40 (s, 1H, CH=N), 8.20–6.99 (m, 12H, Ar-H), 4.18 (s, 3H,OCH_3_); ^13^C-NMR δ: 160.63, 159.13, 149.00, 148.88, 145.83, 143.91, 142.59, 138.99, 132.72, 131.91, 129.88, 127.32, 126.79, 125.79, 123.56, 123.35, 121.99, 120.39, 119.79, 118.98, 116.79, 116.21, 115.49, 114.78, 53.90; Anal. Calcd. for C26H17Cl_3_ N4O (507.21): C 60.65, H 3.70, N 10.69; found C 60.90, H 3.53, N 10.87. 

*7-Chloro-2-methoxy-10–(4-(2′-pyridylidene)aminophenyl)-amino-benzo[b][1,5] naphthyridine* (**4l**). Yield 48%, m.p. 227.2–228.7 °C. ^1^H-NMR δ: 8.75 (s, 1H, Ar-NH), 8.41(s, 1H, CH=N), 8.80–7.05 (m, 13H, Ar-H), 4.18 (s, 3H,OCH_3_); ^13^C-NMR δ: 160.69, 159.23, 149.09, 148.78, 145.85, 143.92, 142.59, 138.99, 132.76, 131.93, 129.88, 127.35, 126.69, 125.81, 123.66, 123.36, 121.98, 120.49, 119.89, 118.45, 116.80, 116.20, 115.38, 114.78, 53.91; Anal. Calcd. for C25H18ClN5O (440.26): C 62.76, H 3.79, N 14.40; found C 62.70, H 3.94, N 14.42. 

*7-Chloro-2-methoxy-10–(4-(3′-pyridylidene)aminophenyl)-aminobenzo[b][1,5]-naphthyridine* (**4m**). Yield 41%, m.p. 190.1–190.2 °C. ^1^H-NMR δ: 8.76 (s, 1H, Ar-NH), 8.40 (s, 1H, CH=N), 8.91–7.09 (m, 13H, Ar-H), 4.18 (s, 3H,OCH_3_); ^13^C-NMR δ: 160.67, 157.97, 148.73, 147.91, 142.91, 141.22, 140.55, 135.07, 130.75, 130.68, 129.88, 127.32, 126.79, 125.79, 123.56, 123.35, 121.99, 120.39, 119.79, 118.98, 116.79, 116.21, 115.49, 114.78, 53.90; Anal. Calcd. for C25H18Cl N5O (440.26): C 68.26, H 4.12, N 15.92; found C 68.28, H 4.14, N 15.94. 

*7-Chloro-2-methoxy-10–(4-(4′-(1H-indol-5′-yl)methylene)aminophenyl)-aminobenzo[b][1,5]-naph-thyridine* (**4n**).Yield 45%, m.p. 226.8–227.7 °C. ^1^H-NMR δ: 8.68 (s, 1H, Ar-NH), 8.40 (s, 1H, CH=N), 8.45–7.10 (m, 14H, Ar-H), 6.61 (s, 1H, Pyrrol-NH), 4.18 (s, 3H,OCH_3_); ^13^C-NMR δ: 160.60, 157.98, 148.76, 147.87, 142.96, 141.24, 140.55, 140.33, 139.80, 136.31, 135.76, 130.69, 130.65, 129.89, 127.36, 126.80, 125.78, 123.58, 123.38, 122.01, 120.39, 119.82, 118.98, 116.79, 116.21, 115.47, 114.96, 53.78; Anal. Calcd. for C28H20Cl N5O (478.30): C 69.33, H 4.31, N 14.23; found C 69.33, H 4.31, N 14.23.

*7-Chloro-2-methoxy-10–(4-(4′-(quinoline-2′-yl)methylene)aminophenyl)-aminobenzo[b][1,5]naph-thyridine* (**4o**). Yield 44%, m.p. 219.7–220.9 °C. ^1^H-NMR δ: 9.24 (s, 1H, Ar-NH), 8.40 (s, 1H, CH=N), 9.05–7.15 (m, 15H, Ar-H), 4.18 (s, 3H,OCH_3_); ^13^C-NMR δ: 160.67, 159.21,158.45, 158.21, 157.99, 148.84, 147.68, 142.91, 141.20, 140.59, 135.14, 130.36, 130.23, 129.89, 127.33, 126.76, 125.78, 123.56, 123.35, 121.99, 120.39, 119.87, 118.98, 116.82, 116.71, 115.49, 115.41, 53.92; Anal. Calcd. for C29H20Cl N5O (490.32): C 70.98, H 4.17, N 14.04; found C 71.01, H 4.18, N 14.12.

### 3.5. Biological assays

All analogues were tested against *N. brazilliensis* to evaluate their anti-intestinal nematode activities. These compounds were tested on 15 groups of rats, each containing five rats. Eighty male rats, weighing 70–80 g, were provided by the Shanghai Animal Center (Chinese Academy of Sciences). Rats were kept at the animal facility of our institute. Animals were acclimatized for 1 week before infection, and they had free access to water and food. The life cycle of *N. braziliensis* were maintained in our laboratory. Egg-positive feces were obtained from *N. braziliensis*-infected rats at 12–20 days post-infection, and then used for the cultivation of *N. braziliensis* third stage infective larvae (NbL3) as described previously [21]. For the *in vivo* test, rats were each infected subcutaneously with 300 NaL3, all infected rats were treated with compounds 11 days post-infection. For oral administration, compounds were suspended or dissolved in 7% Tween 80 and 3% alcohol, and the concentrations of each drug were 10 mg/kg. The reference compound was albendazole, and water containing 3% alcohol and 7% Triton X-80 was used as a negative control. The percentage deparasitization was calculated using the following formula:

n/N × 100

where N = average number of total worms found from small intestine, large intestine and feces in the control animals and n = average number of worms found from large intestine and feces in the control animals. Evaluations were based on a percentage scale of 0–100, in which 100 was total kill and 0 was no activity. 

## 4. Conclusions

In summary, various types of substituted benzonaphthyridine derivatives were synthesized and their biological activities towards the *N*. *brazilliensis* were demonstrated. Among these compounds, **4n **produced the highest activity against *N*. *brazilliensis*, with 80.2% deparasitization. The present work suggested that **4n **may be a useful lead compound for anti-intestinal nematode medicine development. Further studies of the structure-activity relationships around the designed compounds are underway.
